# A Decade of Biochemical and Structural Studies of the DNA Repair Machinery of *Deinococcus radiodurans*: Major Findings, Functional and Mechanistic Insight and Challenges

**DOI:** 10.1016/j.csbj.2016.04.001

**Published:** 2016-04-27

**Authors:** Joanna Timmins, Elin Moe

**Affiliations:** a Université Grenoble Alpes, Institut de Biologie Structurale, F-38044 Grenoble, France; b CNRS, IBS, F-38044 Grenoble, France; c CEA, IBS, F-38044 Grenoble, France; d The Norwegian Structural Biology Centre (NorStruct), Department of Chemistry, UiT the Arctic University of Norway, N-9037 Tromsø, Norway; e Instituto de Tecnologia Quimica e Biologica (ITQB), Universidade Nova de Lisboa, Av da Republica (EAN), 2780-157 Oeiras, Portugal

## Abstract

The *Deinococcus radiodurans* bacterium is extremely resistant to ionising radiation and desiccation and can withstand a 200-fold higher radiation dose than most other bacteria with no loss of viability. The mechanisms behind this extreme resistance are not fully understood, but it is clear that several factors contribute to this phenotype. Efficient scavenging of reactive oxygen species and repair of damaged DNA are two of these. In this review, we summarise the results from a decade of structural and functional studies of the DNA repair machinery of *Deinococcus radiodurans* and discuss how these studies have contributed to an improved understanding of the molecular mechanisms underlying DNA repair and to the outstanding resistance of *Deinococcus radiodurans* to DNA damaging agents.

## 1 Introduction

*Deinococcus radiodurans* is a Gram-positive, pink pigmented, spherical bacterium, which displays an outstanding resistance to a wide range of DNA damaging agents (desiccation, ionising radiation, reactive oxygen species (ROS), toxic chemicals *etc.*), making it a very robust bacterium [1], [2], [3]. Under extreme conditions, *D. radiodurans* stops growing, but can efficiently recover or ‘resurrect’ and replicate once ordinary growth conditions are restored [4]. Recent studies have revealed that this polyextremophilic phenotype relies largely on a highly resistant proteome and an efficient DNA repair system, rather than on the ability of *D. radiodurans* to protect its genome (recently reviewed in [5]). *D. radiodurans* has indeed been shown to possess several efficient and unusual anti-oxidant systems, notably increased ROS-detoxifying and scavenging activities, that are essential for the protection of its proteome from radiation and oxidation damage [6], [7], [8].

In contrast, based on genome analysis, *D. radiodurans* exhibits, in appearance at least, a mostly ‘classical’ prokaryotic DNA repair machinery, consisting of the base excision repair (BER), the nucleotide excision repair (NER), the mismatch repair (MMR) and double-strand break (DSB) repair pathways [9], [10]. A majority of the proteins from these pathways are indeed widespread and conserved in other bacteria, suggesting that the DNA repair machinery of *D. radiodurans* in itself is most likely not sufficient to confer radiation resistance. But when taking a closer look, several unusual features distinguish *D. radiodurans* from radiation-sensitive bacteria [9]. *D. radiodurans*' genome encodes a large number of DNA glycosylases involved in BER, two Dps proteins that contribute to DNA protection [11], [12], [13], two single-stranded DNA binding (SSB) proteins (SSB and DdrB) and many hypothetical proteins, some of which may be involved in DNA repair. *D. radiodurans* also possesses two variants of the UvrA (UvrA1 and UvrA2) and MutS (MutS1 and MutS2 [14]) proteins involved respectively in NER and MMR, and a number of *Deinococci*-specific proteins (*e.g.* DdrA, DdrB, DdrC, DdrD, IrrE and PprA) that are highly expressed following radiation or desiccation and appear to be playing important roles in DNA damage response [2], [15], [16], [17], [18], [19]. Interestingly, *D. radiodurans* is also missing several key DNA repair proteins, notably photolyases and translesion polymerases, but also a DNA dioxygenase (AlkB) and a RecBC recombinase [9].

Over the past decade, the DNA repair machinery of *D. radiodurans* has been the focus of many genetic, biochemical, biophysical and structural studies, which have greatly contributed to our understanding of DNA repair processes, providing critical insight into the basic molecular mechanisms underlying DNA damage recognition and repair in prokaryotes. Many of these studies have also revealed unanticipated differences between *D. radiodurans* proteins and their homologues from radiation-sensitive organisms indicating possible strategies devised by *D. radiodurans* to efficiently repair its DNA. A good illustration of this comes from the study of *D. radiodurans* SSB proteins. As mentioned above, one of the particularities of *D. radiodurans* is that its genome encodes two types of SSB proteins [9]: a ‘classical’ SSB protein and a new variant, called DdrB [20]. Although SSB proteins are not directly involved in DNA repair (and will therefore not be discussed further in the subsequent sections), they are key components of many DNA-associated processes and in *D. radiodurans* both of these SSB proteins play important roles in protecting ssDNA intermediates and acting as protein scaffolds during the recovery phase following major DNA damage. The ‘classical’ *Deinococcus–Thermus* SSB protein actually differs from the prototypical SSB from *Escherichia coli* since it is composed of two non-identical OB-fold domains (resulting from gene duplication) connected by a β-hairpin linker and assembles as homo-dimers and not as homo-tetramers [21]. In spite of the asymmetry produced by the two tandem OB-fold domains within SSB, the four OB-folds of *D. radiodurans* SSB dimers bind ssDNA in a very similar way to tetrameric *E. coli* SSB [22]. DdrB, on the other hand, is a *Deinococci*-specific protein that is highly expressed following DNA damage and exhibits many of the usual SSB functions (binds tightly to ssDNA and modulates the formation of RecA filaments). However, it forms pentameric, ring-like assemblies [20], very different from ‘classical’ SSBs. The crystal structure of *Deinococcus geothermalis* DdrB revealed that it does not adopt a typical OB-fold, but instead represents the founding member of a new class of SSB proteins [23], which may well provide *D. radiodurans* with a new means of responding to high levels of DNA damage.

The aim of this review is thus to provide a comprehensive overview of the numerous studies of the DNA repair machinery of *D. radiodurans*, to summarise our current knowledge in terms of structural and functional characterisation of these proteins and to relate these findings to *D. radiodurans*' outstanding resistance to DNA damaging agents.

## 2 Structural Studies of *D. radiodurans* DNA Repair Proteins

*D. radiodurans* proteins have proven to be well suited for structural studies. Table 1 provides an overview of the structural studies performed in the recent years on *D. radiodurans* proteins that are known to be directly involved in one or several of the above mentioned DNA repair pathways. More detailed descriptions of the structures of these key proteins and related functional studies are provided in the following sections.

## 3 Base Excision Repair

The Base Excision Repair (BER) pathway is highly conserved across all kingdoms of life. It is responsible for the repair of deaminated, alkylated and oxidised bases, spontaneously formed abasic sites and single-stranded breaks in DNA. BER is initiated by DNA glycosylases, which are either monofunctional or bifunctional and exhibit specificity for a subset of damaged bases. Both groups of enzymes recognise the damaged bases, flip them out of the DNA and into the catalytic pocket and remove them by hydrolysing the N-glycosidic bond between the base and the sugar phosphate backbone, thereby generating an abasic site (AP-site). In the case of monofunctional glycosylases, this site is further processed by an AP-endonuclease (Exonuclease III or Endonuclease IV), which cleaves the DNA on the 5′ side of the AP-site leaving a 3′-OH and 5′-deoxyribose phosphate (5′-dRP) termini [24]. The 5′-overhang is subsequently removed by a dRP lyase activity of DNA polymerase producing a 5′ phosphate end, which allows the polymerase to incorporate the correct base and the DNA ligase to seal the gap. Bifunctional glycosylases, on the other hand, cleave the DNA on the 3′ side of the AP-site after removal of the damaged or modified base, leaving a 3′-unsaturated aldehyde or 3′-phosphate and a 5′-phosphate group [25], [26]. The aldehyde or phosphate ends are further processed by AP-endonucleases in order to allow the DNA polymerase and DNA ligase to introduce the correct base and seal the gap.

The genome sequence of *D. radiodurans*[27] revealed that it contained a relatively high number of DNA glycosylases compared to other bacteria (Table 2). Five genes encoding uracil DNA glycosylases (UDGs) were reported: one uracil-DNA N-glycosylase (drUNG), one mismatch specific uracil DNA N-glycosylase (drMUG), one thermophilic UNG (drTmUNG) and two hypothetical UDGs (encoded by ORFs DR_0022 and DR_1663). The two latter are relatively small (~ 15 kDa) and display low sequence identity with other UDGs, but have however been annotated as UDGs. *D. radiodurans*' genome also encodes two 3-methyladenine DNA glycosylases II (drAlka1 and drAlka2), but no 3-methyladenine DNA glycosylase I (Tag) and AlkB (dioxygenase) enzymes. DrAlkA1 has high homology to eukaryotic AlkAs, while DrAlkA2 has been described to be of bacterial origin. Of the oxidation damage glycosylases, *D. radiodurans* possesses one formamidopyrimidine DNA glycosylase (MutM/Fpg), one A/G specific adenine DNA glycosylase (MutY) and three Endonuclease III enzymes (EndoIII-1, -2 and -3), but no Endonuclease VIII/Nei. As for the end processing, base insertion and ligation enzymes, only one AP-endonuclease (Exonuclease III/DR_0354) has been identified in addition to DNA polymerase I (DR_1707) and two DNA ligases (LigA/DR_2069 and LigB/DR_B0100).

**Fig. 1:**
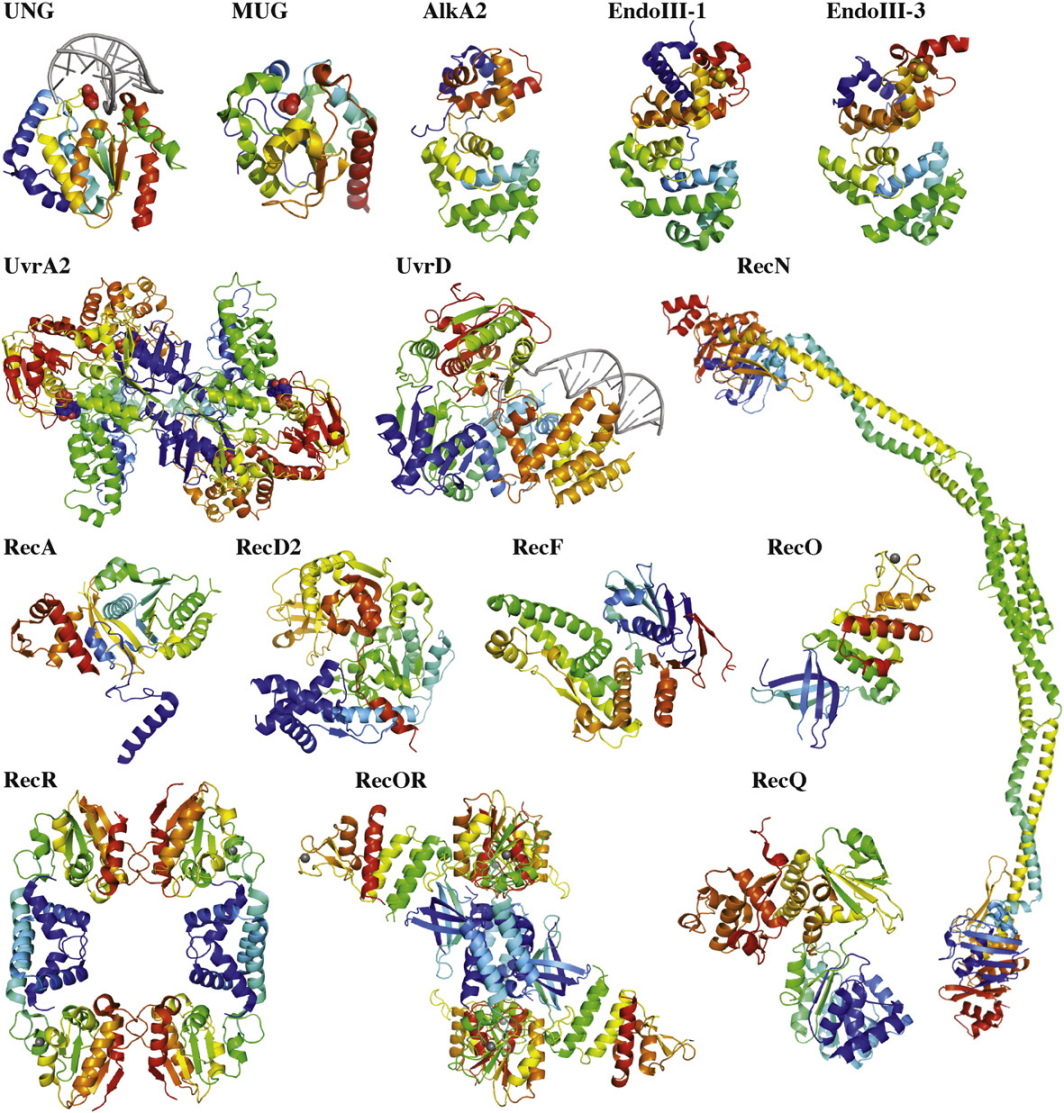
Illustration of the structures of *D. radiodurans* proteins involved in DNA repair processes. All structures are presented in rainbow colours (N-termini in blue and C-termini in red). The following PDB codes were used to prepare the figures using Pymol [126]: UNG (4uqm), MUG (2c2p), AlkA2 (2yg8), EndoIII-1 (4unf), EndoIII-3 (4uob), UvrA2 (2vf7), UvrD (4c2v), RecN (4aby; 4abx; 4ad8), RecA (1xp8), RecD2 (3e1s), RecF (2o5v), RecO (1w3s), RecR (1vdd), RecOR (4jcv) and RecQ (4q47).

Of these BER enzymes from *D. radiodurans*, a large majority have been produced recombinantly and most of the DNA glycosylase enzymes have been assayed for DNA glycosylase and/or lyase activity. So far, only two of these enzymes have no detectable activity (the hypothetical UDG, DR_0022 and drEndoIII-3; Table 2). Structural studies have also been performed on several of these enzymes (Fig. 1) and at present crystal structures of drUNG (in absence and presence of DNA), drMUG, drAlkA2 and drEndoIII-1 and -3 have been determined [28], [29], [30], [31], [32].

Uracil DNA glycosylases are the best-studied DNA glycosylases. They remove uracil, formed as a result of deamination of cytosine [33] or mis-incorporated during replication [34], from DNA. The drUNG structure was determined to a resolution of 1.8 Å [28] and showed high similarity to previously determined structures of human [35], herpes simplex virus [36], *E. coli*[37] and Atlantic cod [38] UNGs. However, drUNG was shown to possess a very high catalytic efficiency on uracil containing substrate (mainly U:A and U:G base pairs) compared to human UNG, which was attributed to a high substrate affinity caused by a cluster of positively charged residues close to the DNA binding site [28]. The recent high-resolution co-crystal structure of drUNG-DNA (Fig. 1) confirmed a strong enzyme-substrate interaction caused by a high number of long-range electrostatic interactions at the protein-DNA interface [29].

Mismatch specific uracil DNA N-glycosylases (MUG) also remove uracil from DNA, but have been shown to possess high specificity for U:G base pairs and little activity on U:A and ssU substrates compared to UNG [39], [40]. The crystal structure of drMUG (Fig. 1) showed high similarity to the previously determined structure of *E. coli* MUG [30], [39]. However, its substrate specificity was found to be broader: it is able to process A:U and ssU, as well as the ‘classical’ MUG substrate G:U [40]. In addition, a novel catalytic residue was identified, Asp93, which had not been observed before and based on a phylogenetic analysis, the enzyme was classified into a new class of MUG/thymidine DNA glycosylase family MUG2 [30].

No structural information is so far available for the remaining UDGs from *D. radiodurans*, however the activity of drTmUDG and the two hypothetical UDGs (DR_0022 and DR_1663) have been assessed [41]. DrTmUDG was found to efficiently remove uracil from single-stranded DNA (ssU) and also to a lesser extent from U:G and U:A base pairs. The hypothetical UDG encoded by DR_0022, in contrast, shows no detectable UDG activity. The second hypothetical UDG encoded by DR_1663 has also been expressed and purified and preliminary results from activity measurements on uracil containing oligonucleotide substrates (U:A, U:G and ssU) indicate no uracil excision activity for this enzyme (Moe's laboratory, unpublished data). Based on inhibitory studies of UDG activity in *D. radiodurans* extracts using the DNA glycosylase inhibitor (Ugi), drUNG was proposed to represent the major UDG activity of *D. radiodurans*. This is in agreement with a comparative study of drUNG and drMUG, in which drUNG displayed 2600 times higher specific activity on a uracil containing substrate than did drMUG [30].

3-methyladenine DNA glycosylases II (AlkA) are monofunctional glycosylases with broad substrate specificity for alkylated bases, *e.g.* 3-methyl adenine (3meA), 7-methyl guanine (7meG) 1,N^6^-ethenoadenine (εA) and hypoxanthine (Hx) [42], [43], [44] and belong to the Helix-hairpin-Helix (HhH) superfamily of DNA glycosylases [45]. Both *D. radiodurans* AlkA genes have been cloned and subjected to expression test, however no expression of the eukaryotic-like drAlkA1 was observed (Moe's laboratory, unpublished data). In contrast, drAlkA2 was expressed in amounts sufficient for purification and crystallisation purposes and its crystal structure was determined by experimental phasing (Fig. 1). It revealed that the conserved N-terminal domain observed in other bacterial AlkA structures [46] is absent in the *D. radiodurans* enzyme [31]. The core structure, composed of a two helical bundle HhH, is however similar, but with a wider DNA binding cleft. Activity assays revealed that drAlkA2 can efficiently process the common 3meA and 7meG AlkA substrates, but not εA and Hx. In addition, it possesses specificity for the AlkB dioxygenase substrates 1-methyladenine and 3-methylcytosine. Interestingly, no gene encoding an AlkB enzyme has so far been identified in the *D. radiodurans* genome, which may explain why drAlkA2 has evolved to be able to process both AlkA and AlkB substrates. Such broad substrate specificity could be accommodated by its wider DNA binding cleft and a highly accessible specificity pocket [31].

In addition to having two AlkAs, *D. radiodurans* possesses three bifunctional Endonuclease III enzymes (drEndoIII-1, -2 and -3), which are responsible for removal of oxidised pyrimidines. The crystal structures of two of the three EndoIIIs (drEndoIII-1 and drEndoIII-3) were determined experimentally (Fig. 1), taking advantage of the [4Fe–4S] clusters in the enzymes, and a reliable homology model of the third member, drEndoIII-2, was generated using *E. coli* EndoIII (> 30% sequence identity) as a model [32]. DrEndoIII-3 had to be N-terminally truncated prior to crystallisation [47], but sequence analysis strongly indicates that the gene was most likely incorrectly annotated during the processing of the genome sequencing data [9]. The overall structures of drEndoIIIs, consisting of two all α-helical domains characteristic of enzymes from the HhH-GPD superfamily, are very similar to previously determined structures [48], [49]. Several differences were nonetheless observed. Both drEndoIII-1 and -3 possess an additional helix αX inserted in domain 2 just before the conserved HhH motif. Also, compared with previous structures, the DNA binding cleft seems to be more open in the case of drEndoIII-1 and instead more closed and thus less accessible for drEndoIII-3. There are also several critical substitutions close to the active site in the case of drEndoIII-1 and in the DNA binding loops of drEndoIII-3 that could explain the differences observed in terms of enzymatic activity and substrate specificity. Such measurements have indeed shown that drEndoIII-2 is a robust enzyme with strong bifunctional activity against the typical EndoIII substrate, thymine glycol (Tg), while drEndoIII-1 only exhibits weak glycosylase and lyase activities on Tg-containing DNA. In the case of drEndoIII-3, no enzymatic activity has so far been detected on common EndoIII substrates, even though the catalytic residues are conserved both in sequence and positioning in the structure [32]. At present, it is still unclear why *D. radiodurans* possesses these three EndoIII enzymes. Having three EndoIII proteins with different catalytic efficiencies and potentially different substrate specificities within the cell most likely contributes to an improved DNA repair repertoire to survive oxidative DNA damage caused by either radiation or desiccation. This hypothesis is supported by the observation that a majority of the members of the *Deinococci* species also possess three EndoIIIs.

So far no crystal structures have been reported of the two other oxidation damage repair glycosylases (MutY and MutM; Table 2) from *D. radiodurans*, however some biochemical characterisation of these enzymes has been performed. The A/G specific adenine DNA glycosylase, MutY, of *D. radiodurans* (drMutY) has been cloned, expressed and characterised, and was found to possess adenine glycosylase activity towards classical MutY substrates with A:G, A:C and A:8oxoG (7,8-dihydro-8-oxoguanine) base pairs like its homologue from *E. coli*[50]. *D. radiodurans* MutM/Fpg (drMutM) has also been characterised and similarly to *E. coli* MutM/Fpg (ecMutM) was found to excise both 2,6-diamino-4-hydroxy-5*N*-methylformamidopyrimidine (Fapy) from DNA paired with both guanine and adenine and to a lesser extent 8oxoG paired with guanine [51]. However, while ecMutM excises these three substrates with similar efficiencies, drMutM was found to prefer Fapy as a substrate [51].

Also, no structural information is available for the downstream end processing, base incorporation and ligation enzymes of *D. radiodurans*, however several of these have been produced and characterised [52], [53]. The unique AP endonuclease (Exonuclease III) of *D. radiodurans* (drExoIII) has been produced recombinantly and preliminary data indicate that the enzyme possesses classical Exonuclease III activity (Moe's laboratory, unpublished data). Originally only one DNA ligase (NAD dependent drLigA) was identified in the genome of *D. radiodurans* (DR_2069) [9], and only later a second gene encoding a divergent ATP-dependent DNA ligase (drLigB/DR_B0100) was discovered [52]. Characterisation of these enzymes revealed that drLigA is a functional DNA ligase on its own [52], while drLigB is only active as a ligase in complex with a hypothetical protein (DR_B0098) encoded by the LigB operon and a pleitropic protein promoting DNA repair enzyme (PprA) [53]. It was also shown that deleting the *ligB* gene makes the LigB operon non-functional and results in loss of DNA damage tolerance [53]. No studies of the DNA polymerase from *D. radiodurans* have so far been reported.

These various studies of *D. radiodurans* BER enzymes have contributed to a better understanding of the fundamental processes leading to repair of damaged bases *via* the BER pathway, but have also revealed that most *D. radiodurans* DNA glycosylases either exhibit increased enzymatic efficiency (*e.g.* drUNG) and/or broader substrate specificity (*e.g.* drMUG and drAlkA2), or are members of extended families (*e.g.* drAlkAs, drUDGs and drEndoIIIs) (Table 3).

## 4 Nucleotide Excision Repair

Bacterial NER is mediated by the sequential action of four proteins [54]. UvrA, acting as a dimer together with UvrB, is responsible for DNA damage recognition. UvrA proteins on their own have been shown to bind preferentially to damaged DNA [55], [56], [57]. After damage recognition, UvrA dissociates from the DNA, while UvrB forms a stable pre-incision complex upon sites of DNA damage. UvrC subsequently binds to the UvrB-DNA complex and incises the damaged DNA strand first on the 3′ side, then on the 5′ side of the lesion [58]. The resulting 12 to 13 nucleotide fragment containing the damaged DNA is released by a DNA helicase, UvrD, and the gap is then filled by DNA polymerase I and ligase.

The genome of *D. radiodurans* encodes for a complete NER pathway: UvrA1 (DR_1771; drUvrA1), UvrB (DR_2275; drUvrB), UvrC (DR_1354; drUvrC) and UvrD (DR_1775; drUvrD). DrUvrD has been shown to be involved in several DNA repair pathways [59] and will thus be discussed below in Section 6 dedicated to DNA repair helicases. In addition, a gene encoding for a second, Class II UvrA protein (DR_A0188; drUvrA2) can be found. Class II UvrAs, also known as UvrA2, are found in many bacteria living in harsh environments and show a high degree of sequence similarity to UvrA1, but are missing the UvrB interaction domain. The precise role of UvrA2 remains unclear, but there is evidence that UvrA2s play a role in DNA repair and tolerance to DNA damaging agents, such as UV or chemical treatment [60], [61], [62]. The crystal structure of the ADP-bound dimeric drUvrA2 was solved to 2.3 Å resolution (Table 1 and Fig. 1) [57]. This structure, together with biochemical and mutational studies of drUvrA2, allowed the authors to propose a model for UvrA binding to DNA, which was later validated by the crystal structure of *Thermotoga maritima* UvrA bound to duplex DNA [63]. The overall structures of UvrA1 and UvrA2 proteins are very similar [57], [63], [64]. UvrAs form head-to-head dimers consisting of a saddle-shaped core composed of four nucleotide-binding domains (NBD). Two or three zinc-binding domains (3 in the case of UvrA1 proteins and 2 in the case of UvrA2 proteins) are inserted within these NBDs: the UvrB interacting domain (in UvrA1 proteins only) and a large insertion domain (ID) are inserted into the N-terminal NBD, while a shorter zinc-finger motif is inserted into the C-terminal NBD. Duplex DNA binds to a large positively charged groove on the concave surface of the UvrA dimers and both the ID and zinc-finger motifs appear to be essential for DNA damage recognition and binding by UvrAs.

At present, no structural information is available for drUvrB and drUvrC proteins. Regarding the interactions between Uvr proteins, unlike their *Geobacillus stearothermophilus* homologues [65], [66], drUvrA and drUvrB proteins do not appear to form a stable complex *in vitro* (Timmins' laboratory, unpublished data), indicating that the affinities and/or stabilities of these complexes might vary from one organism to another.

Additional enzymes are found in bacteria for the repair of UV-induced DNA damage [67]. Interestingly, several of these widespread enzymes, such as photolyases or FLAP endonucleases, are missing in *D. radiodurans* and only the UvsE-dependent excision repair pathway (UVDE) involving the UV damage endonuclease (drUvsE) is found in *D. radiodurans*. In this pathway, UvsE introduces a nick immediately 5′ to a UV-lesion that is then processed by other downstream proteins. The respective contribution of the NER and the UVDE pathways to eliminate pyrimidine dimers from UV-irradiated *D. radiodurans* DNA has been evaluated: UVDE efficiently removes both cyclobutane pyrimidine dimers (CPDs) and pyrimidine (6–4) pyrimidone dimers (6–4 PPs), whereas NER seems more specific for 6–4 PPs [61]. Moreover, inactivation of the two pathways does not completely abolish the ability to eliminate CPDs and 6–4 PPs from DNA suggesting the possible presence of a third back-up pathway [61]. So far, there has been no structural or functional characterisation of drUvsE, but the crystal structure of a close homologue, *Thermus thermophilus* UvsE, reveals that it is structurally similar to *E. coli* Endonuclease IV [68].

## 5 Double-Strand Break Repair

DNA double-strand breaks (DSB) represent a serious threat to genome stability and cell survival and can lead to major genome rearrangements. In bacteria, the predominant repair pathway for DSBs is homologous recombination (HR). HR repairs DSBs accurately in a step-wise manner by using information from an intact homologous template. HR takes place in five major stages: (i) DSB recognition, (ii) DNA end processing, (iii) RecA loading, (iv) strand invasion and branch migration and (v) Holliday junction resolution [69]. In *D. radiodurans*, HR has been shown to be preceded by an extended synthesis-dependent strand annealing (ESDSA) process to allow rapid reconstitution of an intact genome following ionising radiation [4], [59]. Over the past decade, the biochemical and structural studies of *D. radiodurans* proteins involved in ESDSA and HR (Table 1 and Fig. 1) have significantly contributed to a better understanding of the molecular mechanisms underlying DSB repair.

DSB recognition is a poorly characterised process in bacteria. In eukaryotes, the central DSB response factor is the MRN complex, consisting of Mre11, Rad50, and Nbs1 [70]. This multifunctional complex triggers the cellular response to DNA damage, prepares the DNA ends for subsequent strand exchange processes *via* its multiple nuclease activities, and is implicated in the tethering of DNA ends and chromatids [71]. Structural homologues of Mre11 and Rad50 are found in all three kingdoms of life, but their roles do not appear to be conserved. Instead, in bacteria there is now increasing evidence that the RecN protein, a member of the SMC (Structural Maintenance of Chromosomes) family, plays an essential role in DSB recognition in several bacterial species [72], [73], [74], [75], [76]. *D. radiodurans* RecN (drRecN) has been the focus of several biochemical studies in the past years [75], [77] and its quasi-atomic structure based on crystallographic and small-angle X-ray scattering (SAXS) data was determined in 2012 (Table 1 and Fig. 1), representing the first complete structure of a member of the SMC protein family [75], [78], [79]. DrRecN consists of a Rad50-like globular *Head* domain, in which the N- and C-terminal domains fold together, and a short and rigid anti-parallel coiled-coil region that differs from the long, flexible coiled-coil regions found in the well-studied condensin or cohesin SMC proteins [80]. DrRecN and its isolated *Head* domain were shown to possess weak ATPase activity that is stimulated by DNA binding. In addition, intact drRecN exhibits cohesin-like activity, whereby it stimulates intermolecular ligation of linear plasmid DNA [77], [79]. The structural characterisation of drRecN revealed that drRecN assembles as a dimer. Unexpectedly, this dimerisation was shown to occur through its coiled-coil region directly, in contrast with other SMC family members that possess a hinge domain implicated in dimer formation. Characterisation of a double Walker A/Walker B mutant of drRecN, which exhibits impaired ATPase activity and tight binding to ATP, also revealed that drRecN's *Head* domain could form ATP-dependent dimers [75]. Together, these findings suggested that drRecN might polymerise in an ATP-dependent manner along DNA in proximity to sites of DSBs.

In contrast with the initial step of HR, the processing of the DNA ends and the loading of the RecA recombinase has been the focus of many studies in the past decades and involves either the RecBCD or the RecFOR pathway [81], [82]. Whereas the RecBCD pathway has been shown to be the major DNA recombination pathway in *E. coli*, the RecFOR pathway actually appears to be the more frequent pathway in bacterial genomes [83]. This is notably the case in *D. radiodurans* in which the *recB* and *recC* genes are missing and the *recD* gene (DR_1902) encodes a modified RecD (known as drRecD2) that possesses an extra N-terminal domain compared to the classical RecD protein [9]. Crystal structures of a proteolytic fragment of drRecD2 missing its N-terminal domain were solved in its apo- [84] (Fig. 1) and ssDNA-bound form [85] and revealed that drRecD2 is very similar to *E. coli* RecD, which is only functional when part of a heterotrimeric complex with RecB and RecC [86]. In contrast, drRecD2 alone was shown to possess 5′–3′ helicase activity with a preference for substrates with a 5′-tail [87], [88], but appears to be dispensable for DSB repair [89].

The RecFOR pathway comprises a 5′–3′ ssDNA exonuclease (RecJ), a DNA helicase (RecQ or UvrD, see Section 6) and the RecF, RecO and RecR proteins that act together to facilitate RecA loading onto SSB-coated ssDNA. In *D. radiodurans*, RecJ (drRecJ) that is responsible for the resection of 5′ ends is essential for viability [59], [90], suggesting that it may be involved in cellular processes other than DSB repair. At present, however, little is known regarding the *in vivo* functions, biochemical activities and structural organisation of drRecJ. In contrast, the numerous studies performed over the past decade on the RecFOR system of *D. radiodurans* have provided considerable functional and structural insight into the mode of action of these proteins [59], [91], [92], [93], [94], [95], [96]. Crystal structures of the individual *D. radiodurans* RecF (drRecF), RecR (drRecR) and RecO (drRecO) proteins have been determined [92], [93], [94] and structural information is also available for the *D. radiodurans* RecOR (drRecOR) complex [95], [96] (Table 1).

DrRecF exhibits extensive structural similarity with the ATPase *Head* domain of Rad50 protein (Fig. 1) [92]. ATP binding triggers drRecF dimerisation [92] and ATP hydrolysis triggers dissociation from DNA [97]. DrRecO contains an N-terminal oligonucleotide binding (OB) fold domain, a central helical domain and a zinc-binding domain (Fig. 1) [94], [98]. DrRecO binds both ssDNA and dsDNA likely *via* its OB-fold domain and, potentially, charged surface areas of other domains [94]. RecO interacts with SSB and promotes the annealing of complementary ssDNA strands [98], [99]. The crystal structure of drRecR revealed a tetrameric architecture (Fig. 1), consisting of a dimer of dimers that has been suggested to act as a DNA clamp [93]. These RecR tetramers exhibit two different RecR–RecR interfaces, involving domain swapping of either the N-terminal or the C-terminal domains. More recently, SAXS data revealed that drRecR is actually a dimer in solution [95], as has been observed for several homologues [100], [101], [102], [103]. These drRecR dimers assemble through their N-terminal domains. RecR interacts with both RecF and RecO *in vitro* and both complexes show a significantly increased apparent affinity for ssDNA and dsDNA compared to the individual proteins [95], [103], [104], [105].

At present, in *D. radiodurans* at least, there is no clear evidence for the formation of a trimeric RecFOR complex. The crystal structure of drRecOR complex solved at 3.8 Å resolution revealed a four-to-two stoichiometry between RecR and RecO [96]. drRecR forms a tetrameric ring and the two drRecO molecules are bound on either side of the ring. Unexpectedly, in the crystal structure, the amino acids in drRecR that are important for DNA binding are occluded by drRecO. This structure led to the idea that the drRecOR complex may undergo a conformational change upon DNA binding. A more recent, higher resolution crystal structure of drRecOR revealed that the complex could indeed adopt a more ‘open’ conformation (Fig. 1) in which the inside of the drRecR ring and the OB domains of drRecO are now accessible for binding ssDNA [95]. In this study, mutagenesis and biochemical data, together with molecular dynamics simulations carried out concomitantly on the drRecOR complex with and without ssDNA, allowed the authors to propose a molecular model for how this complex interacts with its DNA substrates and facilitates the loading of RecA onto DNA.

**Fig. 2:**
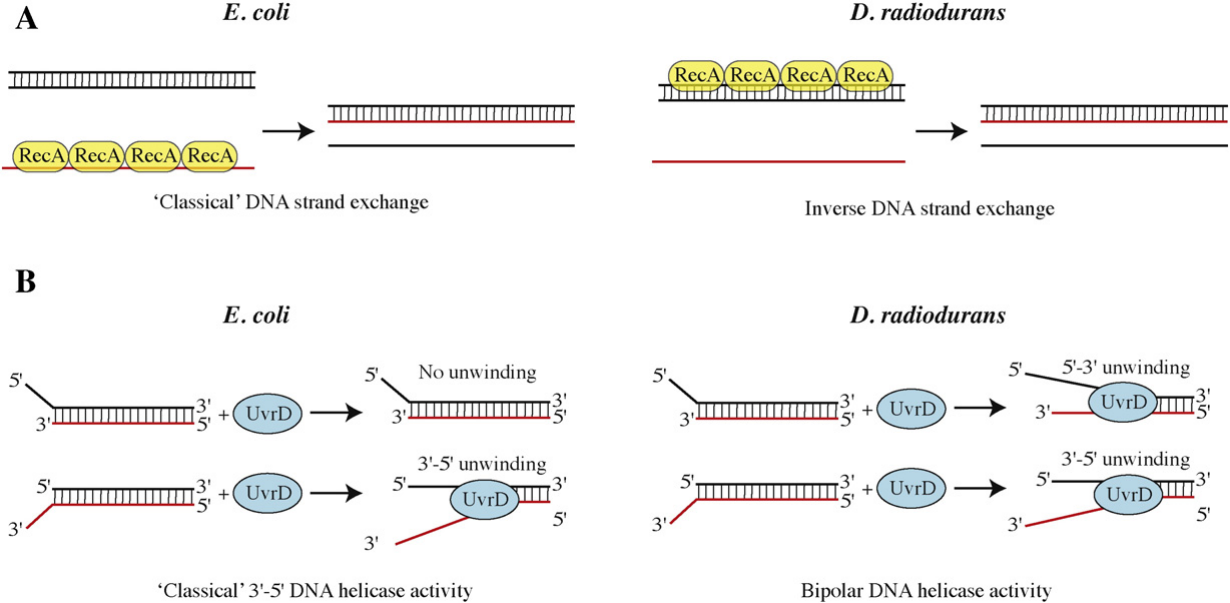
Examples of altered DNA repair processes in *D. radiodurans*. (A) RecA promoted DNA strand exchange in *E. coli vs. D. radiodurans*. Unlike *E. coli* RecA, drRecA assembles on dsDNA. (Figure adapted from [108].) (B) DNA unwinding by the UvrD helicase in *E. coli vs. D. radiodurans*. Unlike *E. coli* UvrD, drUvrD is a bipolar helicase; it unwinds both 3′- and 5′-tailed dsDNA.

Finally, *D. radiodurans* RecA (drRecA) has also been the focus of several studies in the recent years. The crystal structure of drRecA in complex with ATPγS was determined to 2.5 Å resolution (Table 1 and Fig. 1) [106]. DrRecA forms a helical filament in the crystals, as has been observed for several RecA homologues and notably *E. coli* RecA [107]. The overall structures of dr- and *E. coli* RecA are similar, but changes in the relative positioning of the N- and C-terminal domains of drRecA significantly alter the pitch of the helical filament as compared to *E. coli* RecA, thereby producing a compressed, inactive drRecA filament. Functional insight into the key role of drRecA in the repair of hundreds of DSBs in *D. radiodurans* has been provided by the biochemical characterisation of drRecA initially using classical *in vitro* methods [108], [109], [110] and more recently using optical tweezer and single-molecule approaches [111], [112]. Like its *E. coli* counterpart, drRecA forms right-handed helical filaments on DNA, hydrolyses ATP in a DNA-dependent fashion, and promotes strand exchange reactions *in vitro*. Importantly, however, in contrast to other known recombinase enzymes, drRecA polymerises on dsDNA [108], [109] and its polymerisation properties are significantly different from those of *E. coli* RecA [112] (Fig. 2 and Table 3). In both cases, multiple nucleation events are observed, but drRecA filaments form at a much faster rate on dsDNA than do *E. coli* RecA. Interestingly, the length of the drRecA filaments formed on dsDNA are slightly shorter than those obtained with *E. coli* RecA, indicating that drRecA filament formation is a more dynamic process. DrRecA's ability to form numerous short-length patches along the DNA may contribute to its highly efficient repair of hundreds of DSBs in irradiated *D. radiodurans* cells.

## 6 DNA Repair Associated Helicases

Two DNA helicases are known to play major roles in DNA repair pathways: the RecQ helicase and the UvrD helicase, both of which are found in *D. radiodurans* (Table 1). RecQ is a 3′–5′ helicase that has been proposed to function as part of the RecFOR pathway together with RecJ and also to suppress illegitimate recombination by stimulating Holliday Junction branch migration [113]. RecQ helicases are typically composed of a core catalytic domain, consisting of a helicase and RQC (RecQ-C-terminal) domain that are essential and sufficient for the ATPase and DNA unwinding activities of RecQ, and a C-terminal HRDC (Helicase and RNaseD-like C-terminal) domain that is involved in DNA binding. *D. radiodurans* RecQ (drRecQ) is rather unusual in that it possesses three HRDC domains. There is substantial crystallographic and NMR structural information available regarding the various domains of drRecQ [114], [115], [116] (Table 1 and Fig. 1) and a SAXS study of the complete RecQ in its apo- and DNA-bound form was recently reported [117]. These studies reveal that drRecQ adopts an extended conformation that undergoes large-scale conformational changes upon binding to its DNA substrate to adopt a closed state [117]. *In vivo*, the role of drRecQ in the repair of DSBs is unclear, but studies of *recQ* knockout strains indicate that drRecQ, unlike *E. coli* RecQ, is not a main actor of DSB repair in *D. radiodurans*[118], [119], [120] (Table 3).

In contrast, the UvrD helicase is known to play a major role in DNA repair in *D. radiodurans*. A *uvrD* knockout mutant shows significantly reduced resistance to γ-irradiation and impaired DSB repair through ESDSA [118]. UvrD is a 3′–5′ helicase, member of the SF1A helicase superfamily and crystal structures of *E. coli* and *D. radiodurans* UvrD (drUvrD) bound to DNA and ATP analogues have shed light on their shared molecular mechanisms underlying ATP-dependent DNA unwinding (Table 1 and Fig. 1) [121], [122]. Interestingly, however, analysis of the DNA unwinding properties of drUvrD revealed that it could unwind both 3′- and 5′-extended DNA substrates, suggesting that it was a bipolar helicase, unlike other members of the SF1A superfamily, such as *E. coli* UvrD [122] (Fig. 2 and Table 3). Moreover, drSSB was shown to modulate drUvrD's unwinding and translocase activities; on 5′-extended DNA for instance, the presence of SSB favoured the 5′–3′ helicase activity over the 3′–5′ translocase activity. These unusual properties of drUvrD may reflect its implication in diverse DNA repair pathways *in vivo*.

## 7 Summary and Outlook

Since its discovery in the 1950s, *D. radiodurans* has surprised biologists with its extraordinary capacity to survive high doses of ionising radiation and despite decades of research, all its secrets have not yet been unveiled. Following the release of its genome sequence in 1999, many laboratories undertook structural and functional studies of the various proteins encoded by its genome, including those involved in DNA repair. At present, crystal structures of 14 essential DNA repair proteins from *D. radiodurans* have been published (Table 1 and Fig. 1), confirming that *D. radiodurans* proteins appear to be more amenable to structural determination than some of their *E. coli* counterparts and that *D. radiodurans* is a valuable alternative to *E. coli* for the study of fundamental bacterial processes [123]. Difficulties in producing and characterising several repair systems from *E. coli* (notably NER and DSB repair proteins) have been largely overcome with the study of *D. radiodurans* proteins. In particular, all the structural information currently available for the RecFOR system comes from studies of *D. radiodurans* targets. These studies have thus provided new, valuable data that have largely contributed to deciphering some of the molecular mechanisms underlying DNA repair.

Several structural and functional studies of *D. radiodurans* proteins have also revealed that seemingly conserved enzymes actually exhibit differences in structure and/or enzymatic activity. This is particularly striking in the case of BER enzymes that are highly homologous to their *E. coli* counterparts in terms of sequence and structure and yet possess altered substrate specificities or increased catalytic activities (Table 3). Individually, these minor differences may not seem significant, but when added together and combined with the fact that *D. radiodurans* possesses an unusually high number of DNA glycosylases these modifications may well contribute to an enhanced DNA repair efficiency.

The high number of DNA glycosylases may also play an important role in preventing DSB formation. Ionising radiation damages DNA by direct ionisation generating hydroxyl radicals, which in turn introduce single-strand breaks and base modifications into the DNA and eventually lead to the formation of DSBs. Single-strand breaks are also formed during the BER process, as a result of the processing of abasic sites. DNA glycosylases have been shown to stay tightly bound to their reaction products (typically abasic sites; reviewed in [124]), thereby preventing the generation of DSBs and maintaining the integrity of DNA, and thus facilitating its repair at a later stage. One of the three EndoIII variants of *D. radiodurans*, drEndoIII-3, displays no activity on classical EndoIII substrates but does bind tightly to oligonucleotides containing a stable abasic site [32]. The function of such an enzyme may be to protect damaged DNA rather than to repair it.

Taken together, these findings suggest that *D. radiodurans* may have devised a survival strategy that combines efficient protection of its proteome and genome with efficient repair of its DNA. To achieve this, *D. radiodurans* possesses expanded DNA repair protein families, makes use of conserved mechanisms, which in some cases have been tweaked to perform better than in radiation-sensitive bacterial strains, and additional *Deinococci*-specific mechanisms (Table 3), many of which still remain to be characterised.

Finally, these studies also reveal that *in vitro* reconstitution of several DNA repair pathways, notably BER, NER and DSB repair, is at reach using *D. radiodurans* proteins. Such functional assays will provide the scientific community with valuable tools to further decipher DNA repair pathways and in particular the complex cross talk between pathways.

## Figures and Tables

**Table 1:** List of *D. radiodurans* DNA repair proteins for which high-resolution structural data is available. The list includes the name of the proteins, their associated open reading frame (ORF), the repair pathway they are involved in (BER, NER, DSB or MMR), their function and the references to the structural studies. In the case of drUNG, structures of both the apo- and the DNA-bound form have been determined. Structures of the individual drRecO and drRecR proteins and of the drRecOR complex have been determined. In the case of drRecD2 and drRecQ, only partial structures of the proteins have been solved, while the quasi-atomic structure of intact drRecN was assembled using high-resolution crystal structures of three overlapping regions.

Protein	ORF	Repair pathway	Function	Reference
UNG	DR_0689	BER	Uracil DNA glycosylase	[28], [29]
MUG	DR_0715	BER	Mismatch-specific uracil DNA glycosylase	[30]
AlkA2	DR_2584	BER	DNA glycosylase with specificity for alkylated bases	[31]
EndoIII-1	DR_2438	BER	DNA glycosylase with specificity for oxidised pyrimidines	[32]
EndoIII-3	DR_0982	BER	DNA glycosylase (unknown substrate)	[32]
UvrA2	DR_A0188	NER (?)	ATPase; Damage recognition	[57]
UvrD	DR_1775	DSBR, NER, MMR	ATP-dependent SF1A DNA helicase	[122]
RecA	DR_2340	DSBR	Recombinase	[106]
RecD2	DR_1902	DSBR	SF1B DNA helicase	[84]
RecO	DR_0819	DSBR	Recombinational repair protein	[94], [95], [96], [98]
RecR	DR_0198	DSBR	Recombinational repair protein	[93], [95], [96]
RecF	DR_1089	DSBR	Recombinational repair protein	[92]
RecN	DR_1477	DSBR (?)	Recombinational repair protein	[75]
RecQ	DR_1289	DSBR (?)	DNA helicase	[114], [115], [116], [117]

**Table 2:** List of *D. radiodurans* DNA glycosylases.

Base damage	Protein	ORF	Short name	DG activity	Reference
Uracil repair	Uracil-DNA N glycosylase	DR_0689	UNG	Yes	[28], [29]
	Mismatch specific uracil DNA glycosylase	DR_0715	MUG	Yes	[30]
	Thermophilic DNA glycosylase	DR_1751	TmUDG	Yes	[41]
	Putative uracil DNA glycosylase	DR_0022	-	No	[41]
		DR_1663	–	No	(unpublished)
					
Methylation repair	3-methyladenine DNA glycosylase II	DR_2074	AlkA1	Not tested	–
		DR_2584	AlkA2	Yes	[31]
					
Oxidation repair	Endonuclease III	DR_2438	EndoIII-1	Yes	[32]
		DR_0289	EndoIII-2	Yes	
		DR_0928	EndoIII-3	No	
	Formamidopyrimidine DNA glycosylase	DR_0493	MutM	Yes	[51], [125]
	A/G specific adenine DNA glycosylase	DR_2285	MutY	Yes	[50]

ORF: Open reading frame. DG: DNA glycosylase.

**Table 3:** Overview of the different mechanisms used by *D. radiodurans* to enhance the efficiency of its DNA repair machinery.

Mechanism	Protein
Altered substrate specificity	MUG
	AlkA2
	MutM
	
Enhanced catalytic activity	UNG
	
Member of expanded family (> 2 variants)	UDG
	AlkA
	EndoIII
	UvrA
	MutS
	
Different function/properties	LigB (?)
	UvrD
	RecFOR, RecN (?)
	RecA
	RecQ
